# Clinical experience with a shape memory polymer peripheral vascular embolisation plug: a case series

**DOI:** 10.1186/s42155-021-00214-w

**Published:** 2021-03-09

**Authors:** Robert A. Morgan, Ian Loftus, Lakshmi Ratnam, Raj Das, Leto Mailli, Mohamad S. Hamady, Kyriakos Lobotesis

**Affiliations:** 1 Department of Radiology, St. George’s University Hospitals NHS Foundation Trust, London, GB UK; 2 Vascular Institute, St. George’s Healthcare NHS Trust, London, GB UK; 3 Interventional Radiology, Guy’s and St Thomas’ NHS Foundation Trust, London, GB UK; 4 Interventional Radiology, Imperial College-London-St. Mary’s Campus, London, GB UK; 5 Neuroradiology, Charing Cross Hospital, London, GB UK

**Keywords:** Shape memory polymer, Peripheral embolisation, Vascular plug

## Abstract

**Background:**

Shape memory polymers are materials that are manufactured in a certain shape, can be stored in a temporary deformed shape, and then return to – or remember – their original shape upon exposure to external stimuli such as temperature and moisture. This property lends itself to application in endovascular medical devices. Peripheral vasculature embolisation devices incorporating this novel technology have become commercially available and this case series, where the data were collected as part of a post market registry, outlines initial clinical experience with these novel devices.

**Results:**

Eight cases are described in this series. The disease state/conditions for which embolisation was indicated were right common iliac artery aneurysms (*n* = 3), a type II endoleak into the thoracic aorta following thoracic endovascular aneurysm repair (*n* = 1), a left inferior gluteal artery aneurysm (*n* = 1), left internal iliac artery aneurysms (*n* = 2), and a case of splenomegaly, where splenectomy was planned after the embolisation procedure (*n* = 1). Target arteries were 5–10 mm in diameter. In each case, at least one IMPEDE Embolization Plug (IMP-Device) of an appropriate diameter was used. All procedures were technically successful and target vessel thrombosis was achieved in all cases. Follow-up imaging available during the 45–90-day data collection timeframe showed sustained vessel occlusion. This case series includes examples of situations commonly encountered when embolising the peripheral vasculature, namely, the use of one or multiple devices in a single vessel and in combination with the use of other embolic devices (e.g., microcoils, gelatin sponge, and PVA particles) in the same case. There were no adverse events related to the specific use of the device.

**Conclusions:**

This small series illustrates the safety and efficacy of this novel sponge-based embolic device for the embolisation of small and medium sized arteries and further experience will demonstrate the utility of the shape memory polymer devices.

## Background

**Fig. 1:**
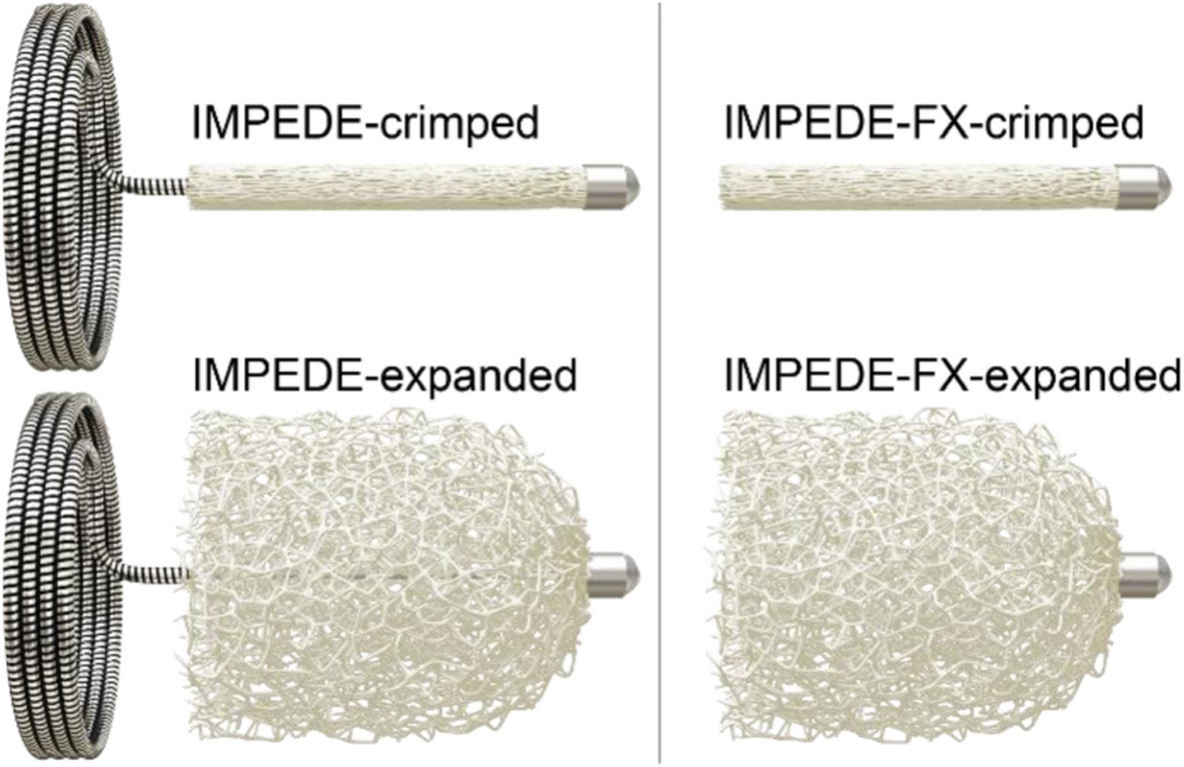
Illustration of the IMPEDE Embolization Plug (left) and IMPEDE-FX Embolization Plug (right) in their crimped and expanded forms. The difference between the devices is that the IMPEDE-FX device does not have an anchor coil. The devices are available with expanded shape memory polymer diameters of 6 mm, 8 mm, and 12 mm. Illustrations are courtesy of Shape Memory Medical

Shape memory polymers can be deformed into a temporary shape and return to – or remember – an original shape upon exposure to external stimuli such as temperature and moisture. The shape memory polymer in the IMPEDE Embolization Plug family (Shape Memory Medical, Santa Clara, California, USA) has been developed with properties that support vessel embolisation. Figure 1 illustrates the IMPEDE Embolization Plug (IMP-Device) and IMPEDE-FX Embolization Plug (IMP-FX-Device. The shape memory portion of the device is crimped for compatibility with catheter delivery and expands to its original space-filling shape once delivered into the warm and aqueous environment of a blood vessel. The expanded shape memory polymer is a porous scaffold that supports thrombus formation throughout the device as the blood penetrates the device upon initial contact. The polymer fabrication and physical properties have previously been described (Landsman et al. 2016). Preclinical work characterised cellular infiltration into the shape memory polymer scaffold and conversion of thrombus to mature collagenous connective tissue over time (Jessen et al. 2020). Furthermore, preclinical work suggests shape memory polymer-based devices support an advanced state of healing, within a designated timeframe, compared to other commonly used vessel embolisation devices (Jessen et al. 2020). Following CE marking of the devices, the manufacturer established a post market study to collect data on their routine clinical use. This case series describes our clinical experience with the novel IMP-Device in the registry study to date.

## Methods

### Study structure, case series

A prospective multicentre registry of the CE marked IMPEDE and IMPEDE-FX Embolization Plug devices, involving no change to standard of care, was approved by London-Hampstead Health Research Authority Research Ethics Committee (Reference IRAS 250759, REC 19/LO/0767). The study is also registered at clinicaltrials.gov (NCT04044443). Participants gave written consent on ethics committee-approved consent forms. Adults (≥18 years) that were candidates for arterial or venous embolisation of the peripheral vasculature were invited to join the study. Those that could not provide written informed consent or were vulnerable persons were excluded. If a patient or planned treatment was outside of the device instructions for use (IFU), the patient was also excluded from the study. The aim of the study is to enroll up to 50 participants and enrollment is ongoing, although hampered by the Covid pandemic. This case series represents the first 8 cases enrolled, all of which used the IMP-Device. Participants were followed through 45 days post procedure to collect follow-up data (including adverse events) during that time. Case 7 was followed for 90 days after a protocol amendment to extend the follow-up period.

### Device use and usability hints and tips

The device was used in accordance with its IFU.

The IMP-Device is available in three sizes: IMP-05, IMP-07, IMP-10 corresponding to expanded plug diameters of 6 mm, 8 mm and 12 mm. The IMP-FX Device is available in three sizes: IMP-FX-06, IMP-FX-08, IMP-FX − 12 corresponding to expanded plug diameters of 6 mm, 8 mm and 12 mm. The recommended vessel diameters for each of the three sizes of each type are 2-5 mm, 4-7 mm and 6-10 mm.

There are two aspects of use that operators should especially note when using these pushable devices, the second being specific to the IMP-Device with the anchor coil. First, the IFU outlines a 1-min working time, defined as the time between when the device enters the catheter and it being deployed into the vessel. Since the polymer begins to slowly expand once in the catheter (because it is in a warm fluidic environment), it may expand enough to cause friction during delivery or even not be able to exit the catheter if the working time is exceeded.

The IMP-05 and IMP-FX-06 can be inserted through an 0.038in catheter. The IMP-07 and IMP-FX-08 can be inserted through a 5Fr guiding catheter or sheath with an internal diameter of 0.055in or more. The IMP-10 and IMP-FX-12 can be inserted through a 6Fr guiding catheter or sheath with an internal diameter of 0.070in or more.

The following description refers to deployment of the IMP-Device. Deployment of the IMP-FX-Device is similar but without the caution regarding an anchor coil, which the IMPFX-Device does not have.

The implant is provided within a short plastic introducer tube. The IMP-Device is advanced into the catheter or delivery sheath by pushing it forward with a stiff 0.035in guidewire.

Once the implant has been pushed out of the plastic introducer into the delivery sheath or catheter, the method of further advancement of the implant varies according its size. The (IMP-5 is pushed through the 0.038in catheter with an 0.035 in. guidewire. The IMP-7 is pushed by the dilator of the 5Fr delivery sheath or guide catheter. The IMP-10 is pushed by the dilator of the 6fr delivery sheath or guide catheter.

It is important not to flush the introducer prior to use or to only flush immediately prior to introduction to the catheter for the same reason (i.e., the working time clock starts upon flushing). Second, the delivery of the anchor coil of the IMP-Device is similar to other coils, however, delivery of the shape memory portion of the device is delivered into the vessel by unsheathing rather than pushing. In practice, this means holding the guidewire steady and retracting the delivery catheter to reveal the shape memory polymer plug. This process ensures the shape memory polymer portion of the device is not pushed into the anchor coil, which would limit its expansion.

### Case follow-up

Cases were followed according to the standard of care throughout the study timeline of 45–90 days post procedure. Any standard of care follow-up imaging during this time was available for evaluation in the study.

## Results

The disease state/conditions for which peripheral vascular embolisation was indicated in each case were right common iliac artery aneurysms (embolized vessels were the right internal iliac arteries (IIA) (cases 1–3), a type II endoleak into the thoracic aorta following thoracic endovascular aneurysm repair (case 4), a left inferior gluteal artery aneurysm (case 5), left internal iliac artery aneurysms (cases 6–7), and splenomegaly (case 8), where a splenectomy was planned after the embolisation procedure. Table 1 summarises brief patient demographics, the target vessels for embolisation, the point of vascular access, the target vessel diameters and of the implanted devices, and any concurrent procedures.

**Table 1:** Case series summary

#	Sex	Age	Target artery	Vascular access	Vessel diameter (mm)	Polymer diameter (mm)	Anchor coil diameter (mm)	Concurrent procedure
1	M	79	R internal iliac	Femoral	10	8 + 12	9 + 13	Iliac stent
2	M	78	R internal iliac	Femoral	8	12	13	EVAR
3	M	69	R internal iliac	Femoral	8	12	13	EVAR and iliac stent
4	M	79	L subclavian	Brachial	10	12	13	–
5	F	79	L inferior gluteal	Femoral	5	6	7	–
6	M	59	L internal iliac	Femoral	9	12	13	–
7	M	66	L internal iliac	Femoral	10	12	13	EVAR
8	F	77	Splenic	Brachial	8	8	9	–

### Cases 1–3 – Right common iliac artery aneurysms

**Fig. 2:**
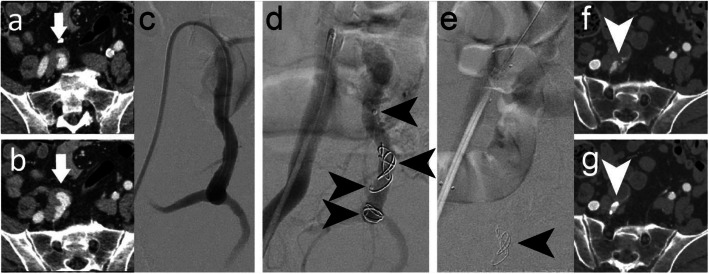
Case 1. **a**, **b** Preprocedural CTA showing a right common iliac artery aneurysm. **c** Pre-embolisation DSA imaging of the right IIA (and aneurysm). **d** 8 mm (distal) and 12 mm diameter (proximal) shape memory polymer plugs were sequentially inserted into the 10 mm diameter right IIA. Arrows indicate the anchor coils and proximal markers of the devices, where the anchor coil is distal to the marker. Shape memory polymer is radiolucent but is between the anchor coil and proximal marker. **e** Post-embolisation DSA imaging where only the proximal device can be seen in this view (arrow). An iliac stent graft was placed after vessel embolisation. **f**, **g** Five-week follow-up CTA showed the vessel was occluded even though a small feeding vessel above the plug (arrow) was perfusing the aneurysm

Figure 2 illustrates embolisation of a right IIA to treat a large common iliac artery aneurysm (case 1). A 6 Fr 45 cm long Destination Sheath (Terumo, Tokyo, Japan) and the sheath of the 6Fr dilator were used for device delivery. An IMP-07 device (diameters: expanded shape memory polymer 8 mm, anchor coil 9 mm) was deployed, followed by an IMP-10 device (diameters: expanded shape memory polymer 12 mm, anchor coil 13 mm), into the 10 mm diameter vessel. Additional embolic material in the form of an IMP-FX-Device was considered. However, stasis occurred with the two devices, and therefore no further embolic material was necessary. An iliac artery stent graft was placed across the origin of the IIA, completing the procedure. Duplex ultrasound assessment prior to discharge of the patient confirmed cessation of flow into the aneurysm. Follow-up computed tomography angiography (CTA) at 5 weeks post procedure showed that the vessel remained occluded, although a very small feeding vessel above the plug was perfusing the aneurysm. No further procedures have been planned or undertaken due to the complexity of any further interventions and the aneurysm size is stable.

**Fig. 3:**
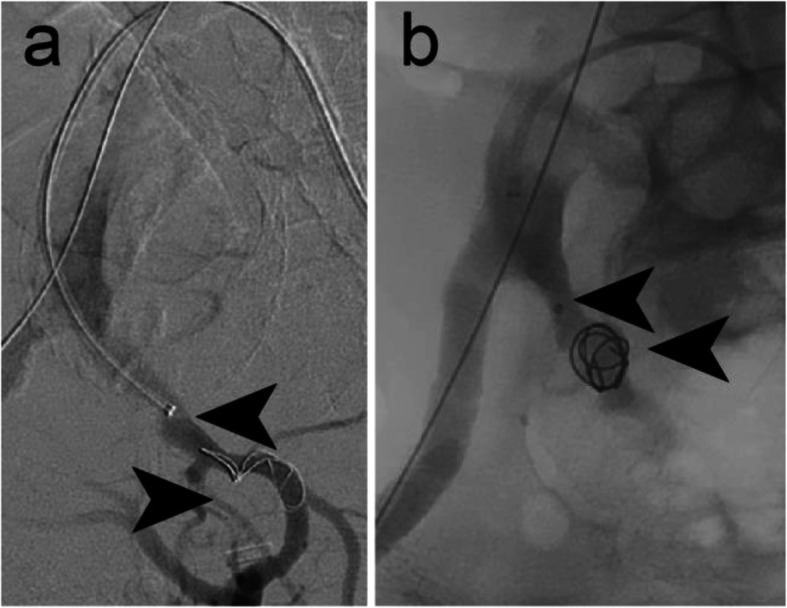
Intraprocedural DSA imaging of **a** case 2 and **b** case 3 (both embolisation of right IIAs) using 12 mm diameter shape memory polymer plugs (one device in each case) in 8 mm diameter right IIAs. Arrows indicate the device anchor coils and proximal markers, where the anchor coil is distal to the marker. Shape memory polymer is radiolucent but is between the anchor coil and proximal marker

Figure 3 shows further intraprocedural angiographic images of right IIA embolisation (cases 2 and 3) to treat common iliac artery aneurysms. In case 2, the patient underwent endovascular aneurysm repair (EVAR) at the same time as vessel embolisation and in case 3, the patient underwent EVAR and right iliac artery stent graft placement at the same time as vessel embolisation. In both cases, a 6 Fr Destination Sheath and the sheath dilator were used to deliver an IMP-10 device (diameters: expanded shape memory polymer 12 mm, anchor coil 13 mm) into each of the 8 mm diameter vessels. Acute vessel embolisation was achieved in both cases. No follow-up imaging to evaluate sustained occlusion was performed within the study timeline. In case 2, the patient experienced postoperative general abdominal pain and in case 3, the patient experienced postoperative buttock claudication and a small groin haematoma. The pain and buttock claudication were managed medically and resolved without sequalae. Buttock claudication is a recognized sequela of IIA aneurysm embolisation, and can be caused by any embolic device used for this procedure. Furthermore, the patients underwent EVAR procedures at the same time as vessel embolisation with associated vascular access requirements.

### Case 4 – Type II endoleak into thoracic aorta

**Fig. 4:**
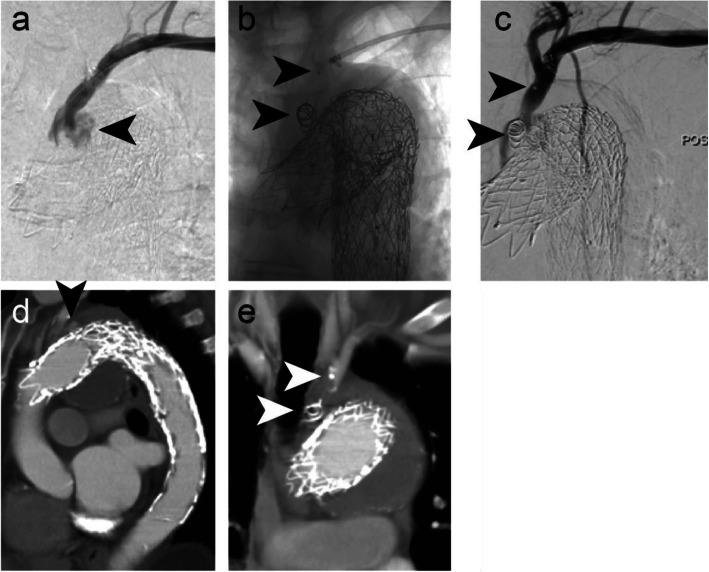
Case 4. **a** Pre-embolisation DSA imaging of a type II endoleak into the thoracic aorta following TEVAR. **b** Intraprocedural DSA imaging following delivery of a 12 mm diameter shape memory polymer plug into the 10 mm diameter left subclavian artery. Arrows indicate the anchor coil and proximal marker. Shape memory polymer is radiolucent but is between the anchor coil and proximal marker. **c** Contrast injection following deployment of the plug. A small endoleak is still present. It should be noted that although complete occlusion ultimately occurs, it may take 10–20 min to occur. **d** Preprocedural CTA shows the type II endoleak. **e** Six-week follow-up CTA confirmed no recurrence of the type II endoleak. Arrows indicate the device anchor coil and proximal marker

Figure 4 illustrates embolisation of a 10 mm diameter left subclavian artery to treat a type II endoleak into the thoracic aorta following TEVAR. An IMP-10 device (diameters: expanded shape memory polymer 12 mm, anchor coil 13 mm) was delivered using a 6 Fr Destination Sheath and the sheath dilator as a pusher via a transbrachial access. Follow-up CTA at 6 weeks post procedure confirmed continued vessel occlusion and no further endoleak into the proximal sac of the descending thoracic aorta. The patient developed a self-limiting mild haematoma related to manual compression after withdrawal of sheath.

### Case 5 – Left inferior gluteal artery aneurysm

**Fig. 5:**
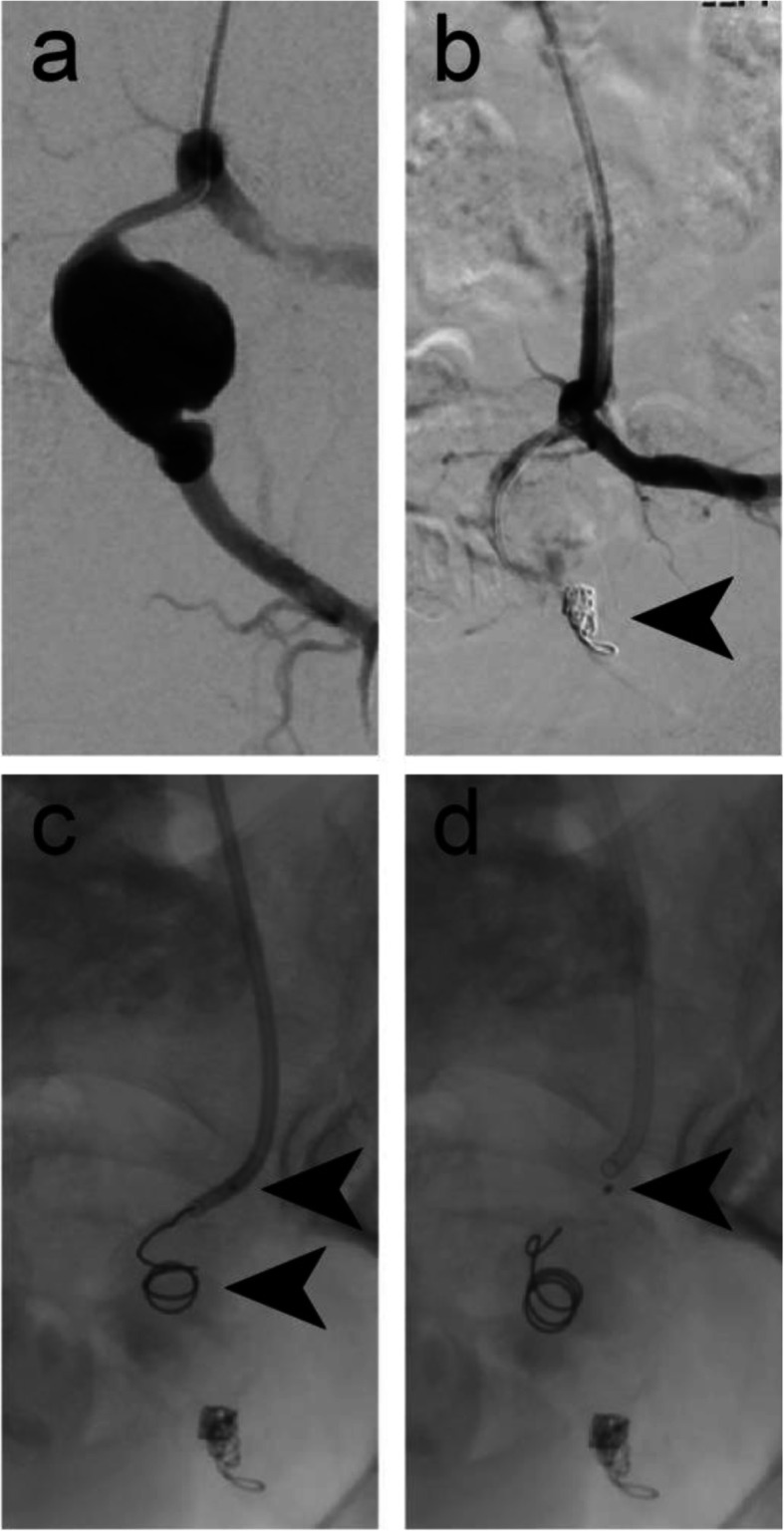
Case 5. **a** Pre-embolisation DSA imaging of the left inferior gluteal artery aneurysm. **b** Microcoils were used for vessel embolisation distal to the aneurysm sac. **c** Intraprocedural DSA imaging showing the anchor coil deployed into the vessel, but immediately prior to deploying a 6 mm diameter shape memory polymer plug into the 5 mm diameter left inferior gluteal artery. Arrows indicate the anchor coil and proximal marker. Shape memory polymer is radiolucent but is between the anchor coil and proximal marker. **d** Intraprocedural DSA imaging immediately following deployment of the plug. Arrow indicates the proximal marker

Figure 5 shows embolisation of a 5 mm diameter left inferior gluteal artery to treat one of the bilateral aneurysms of the participant. In this case, an IMP-05 device (diameters: expanded shape memory polymer 6 mm, anchor coil 7 mm) was delivered using a 4 Fr Glidecath Cobra 2 catheter (Terumo, Tokyo, Japan) and a 180 cm long Bentsen guidewire (W Cook Europe, Bjaeverskov, Denmark). Microcoils were deployed distal to the aneurysm sac and the IMP-Device was used to embolise the vessel proximal to the aneurysm sac. Acute vessel embolisation was achieved. The six-week follow-up CTA showed a sustained vessel occlusion and a decrease in the aneurysm diameter from 26.5 mm to 24 mm.

### Cases 6–7 – Left internal iliac artery aneurysms

**Fig. 6:**
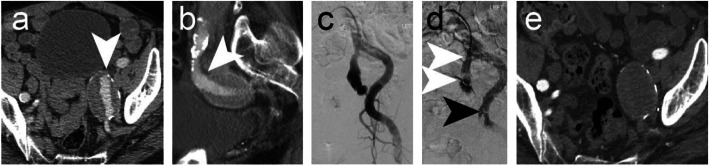
Case 6. Preprocedural CTA showing a left IIA aneurysm - axial (**a**) and sagittal (**b**) sections. **c** Pre-embolisation DSA imaging. **d** Microcoils were used for vessel embolisation distal to the aneurysm sac (black arrow). A 12 mm diameter shape memory polymer plug was deployed into the 9 mm diameter left IIA. White arrows indicate the anchor coil and proximal marker. Shape memory polymer is radiolucent but is between the anchor coil and proximal marker. **e** Six-week follow-up CTA confirmed occlusion of the aneurysm

Figure 6 shows embolisation of a 9 mm diameter left IIA to treat an aneurysm (case 6). An IMP-10 device (diameter: expanded shape memory polymer 12 mm, anchor coil 13 mm) was delivered using a 6 Fr Destination Sheath and the delivery sheath dilator as a pusher. Microcoils were used to embolise distal branches and the IMP-Device was used to embolise the main IIA trunk. Follow-up CTA at 6 weeks post procedure revealed satisfactory occlusion of the aneurysm. In case 7, an IMP-10 device was delivered into a 10 mm diameter left IIA using a 6Fr Destination sheath and the sheath dilator. The patient underwent EVAR during the same procedure. Follow-up imaging duplex ultrasound assessment at 2 weeks post procedure was satisfactory with no residual endoleak.

### Case 8 – Splenomegaly

**Fig. 7:**
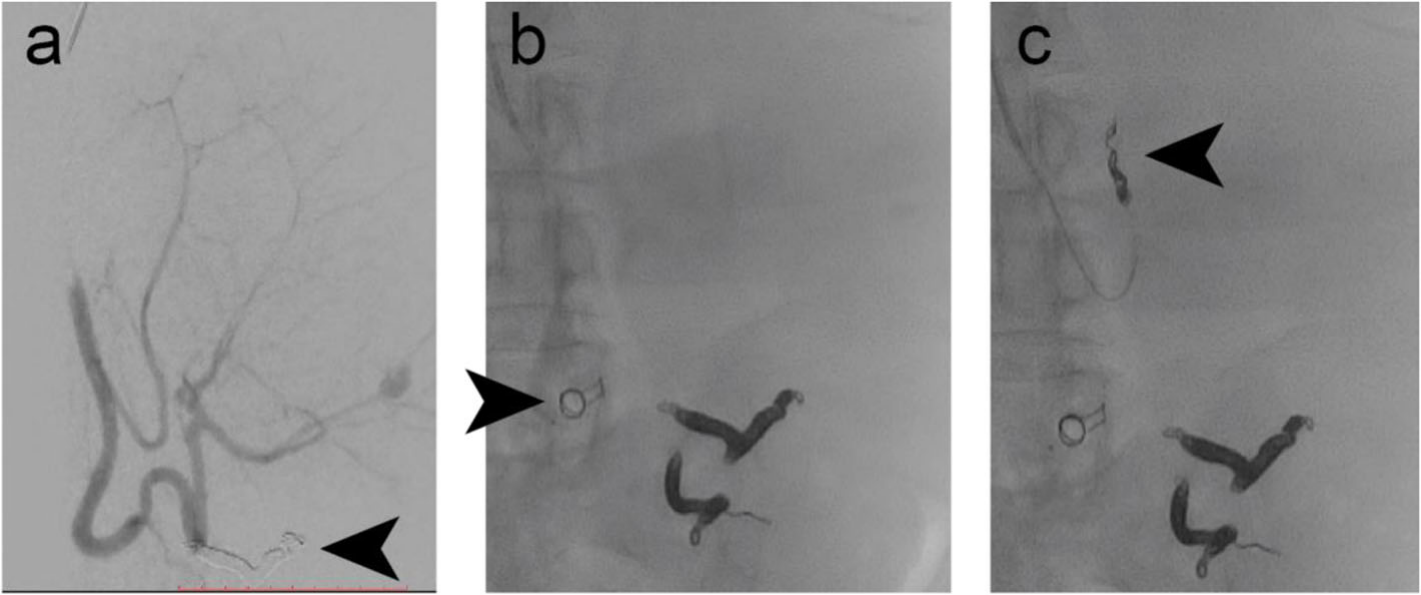
Case 8. **a** Pre-embolisation DSA imaging of the splenic artery. The arrow indicates the result of a prior coil embolisation procedure. **b** An 8 mm diameter shape memory polymer plug was deployed into the 8 mm diameter splenic artery (arrow indicates the anchor coil) after distal packing with gelatin sponge and PVA particles. **c** Microcoil embolisation of another branch completed the case, prior to splenectomy to treat splenomegaly

Figure 7 shows embolisation of an 8 mm diameter splenic artery prior to a planned splenectomy. An IMP-07 device (diameter: expanded shape memory polymer 8 mm, anchor coil 9 mm) was delivered via a 5 Fr delivery sheath and using its dilator as a pusher via a brachial access. Equispon haemostatic gelatin sponge (Equimedical, Zwanenburg, The Netherlands) and Contour PVA particles (500–710 μm) (Boston Scientific, Marlborough, Massachusetts, USA) were used to pack the distal branches prior to vessel embolisation with the IMP- Device. Six Complex Helical-18 coils (Boston Scientific, Marlborough, Massachusetts, USA) were used to embolise a different branch prior to case completion. The splenectomy was performed 2 days later, as planned. The patient developed a haematoma and a small pseudoaneurysm, which significantly decreased in size upon manual compression. This is not an uncommon complication following transbrachial artery access.

## Discussion

The shape memory polymer-based porous embolic scaffold in the IMPEDE Embolization Plug device family is a material new to medical devices, and its properties, particularly its porosity, mechanisms of healing, and lack of radio-opacity, are unique. Peripheral vasculature embolisation is a good initial clinical application of this novel material. As outlined above, the devices performed as expected in the embolisation of small and medium sized arteries. The follow-up imaging showed sustained occlusion of the treated vessels. The IFU states “[Shape memory polymer] undergoes slow degradation with the majority (>90%) of the SMP plug remaining at 30 days in a porcine intravascular model. Near complete degradation was observed in vivo in rat subcutaneous and rabbit intramuscular implants at 180 days.” Longer-term follow-up will show longer-term clinical results, however preclinical work demonstrated the conversion of thrombus to mature collagenous connective tissue as the polymeric scaffold degrades (Jessen et al. 2020).

In terms of practical operations, the 1-min working time of the device is manageable, but catheter choice is an important aspect of case planning. The delivery catheter internal diameters should be in line with the device labeling to avoid potential friction during delivery. The technique of unsheathing the shape memory polymer portion of the device, as outlined above, should be familiar to interventionists. This is an important operational detail to avoid deploying the shape memory polymer into the anchor coil and thereby potentially limiting its expansion. The manufacturer recommends slightly oversizing the device based on the shape memory polymer plug diameter and since the radial force of the shape memory polymer is minimal, this appears reasonable. The anchor coil of the IMP-Device has a landing zone of 2–4.5 cm, depending on the size of the device, and this should be taken into consideration during case planning.

Although the IMPEDE-FX Embolization Plug has not been used in the cases to date, it was considered in case 1. The IMPEDE-FX device offers interventionists the ability to add a large amount of embolic material without adding much artifact in follow-up imaging. The volume of the expanded 12 mm diameter IMPEDE-FX device is approximately 1.25 mL and the proximal radiopaque marker is the only radiopaque material. Case 4, the embolisation of the subclavian artery to treat a type II endoleak following TEVAR, is a reasonable example of how the radiolucency of the shape memory polymer contributes to the clarity of follow-up imaging. It is our understanding that the IMPEDE-FX devices are best used behind another device to avoid potential migration. This other device could be the IMP-Device or a microcoil, for example. Practical use in the ongoing study will result in future examples for evaluation.

The limitations of this study are the limited patients enrolled to date. However, data collection is ongoing and future lessons learned in terms of utility and application of shape memory polymer-based devices will follow.

## Conclusion

All procedures were technically successful and target vessel thrombosis was achieved in all cases. This case series includes examples of situations commonly encountered when embolising the peripheral vasculature, namely the use of one or multiple devices in a single vessel and in combination with the use of other embolic devices (e.g., microcoils, gelatin sponge, and PVA particles) in the same case. Adverse events were few and were those commonly encountered when embolising the peripheral vasculature with any embolic device. Moreover, they were unrelated to the use of this specific device.

In summary, this small series illustrates the safety and efficacy of this novel sponge-based embolic device for the embolisation of small and medium sized arteries and further experience will demonstrate the utility and potential of the shape memory polymer devices.

## Data Availability

Not applicable.

## Abbreviations

CTA: Computed tomography angiography
EVAR: Endovascular aneurysm repair
IFU: Instructions for use
TEVAR: Thoracic endovascular aneurysm repair
